# Revealing the polar nature of a ferroelectric nematic by means of circular alignment

**DOI:** 10.1038/s41598-021-04028-7

**Published:** 2021-12-23

**Authors:** Per Rudquist

**Affiliations:** Department of Microtechnology and Nanoscience, Chalmers University of Technology, 41296 Gothenburg, Sweden

**Keywords:** Liquid crystals, Structure of solids and liquids

## Abstract

The recent discovery of spontaneously polar nematic liquid crystals—so-called ferroelectric nematics—more than a century after the first discussions about their possible existence—has attracted large interest, both from fundamental scientific and applicational points of view. However, the experimental demonstration of such a phase has, so-far, been non-trivial. Here I present a direct method for the experimental verification of a ferroelectric nematic liquid crystal phase. The method utilizes a single sample cell where the two substrates are linearly and circularly rubbed, respectively, and the ferroelectric nematic phase (N_F_) is revealed by the orientation of the resulting disclination lines in the cell.

## Introduction

**Figure 1:**
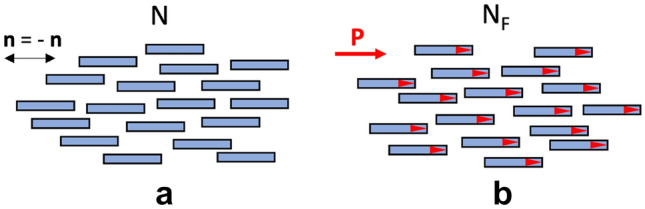
Schematic representations of (**a**) the nematic (N) and (**b**) the ferroelectric nematic (N_F_) phases.

The simplest nematic liquid crystal phase (N, Fig. 1a) is a three-dimensional orientationally ordered liquid. The local average direction of the molecules is denoted by the director **n**, and characteristic for the N phase is that even if the constituent (usually rodlike) molecules may be polar themselves, n is invariant under sign reversal, i.e., **n** = − **n**. The structure and non-polar symmetry of the N phase is well-known since more than a century and the electrooptic effects of nematic liquid crystals are the basis for the multi-billion dollar liquid crystal display technology.

In 2017 Nishikawa et al.^1^ and Mandle et al.^2,3^, reported materials which apparently exhibited nematic phases where locally the sign-invariance of **n** was not valid. Nishikawa et al., reported a phase transition between two fluid nematic phases in the highly polar compound DIO. In the lower temperature phase, they observed macroscopic polar ordering, probed by means of SHG measurements, in response to an applied electric field. They suggested the dipolar molecular associations to be “ferroelectric-like”. Mandle et al. proposed that the local polar order in the material RM734 was driven by spontaneous splay, (splay nematic phase) which resulted a periodic structure, with alternating direction of splay and polarization^2–4^. Later, Sebastián et al., by means of second harmonic generation imaging, could draw the conclusion that the local order in the “splay nematic phase” of RM734 is polar on a macroscopic scale and that the splay and polarization modulation period is 5–10 µm. They also raised the important question whether a homogeneous uniaxial ferroelectric nematic phase can be realized, or if ferroelectric ordering in a nematic phase must be accompanied by orientational deformation^5^.

In 2020, Clark and co-workers reported experimental evidence that the new liquid crystal phase in RM734, first synthesized in 2017 by Mandle, Cowling and Goodby^2–4^, is the ferroelectric nematic (N_F_) phase^6^, cf. Figure 1b. The possible existence of such a spontaneously polar nematic phase, driven by dipolar ordering of molecular dipoles in highly polar materials, was proposed already by Born in 1916^7^ but evidence for its existence has not been presented before. The discovery of the N_F_ phase “opens another chapter in condensed matter science and technology” (Lavrentovich^8^) and has sparked an enormous interest, both from fundamental physics and from applicational points of view.

To understand the mechanisms behind the formation of the new N_F_ phase, and to explore its future application potential, there is an immediate need to design, synthesize and investigate a vast number of similar materials, and rapidly scan these for the N_F_ phase. In this letter I present a surprisingly simple and fast method for direct experimental identification of the N_F_ phase, utilizing only a single sample cell and an optical microscope (or even the bare eye). No external electric or magnetic fields, no second harmonic generation experiments, nor any spectroscopic methods are needed. The study solely treats the experimental identification of the N_F_ phase, with no ambition at this stage, to further elaborate on the mechanisms behind the formation of the N_F_ phase.

**Figure 2:**
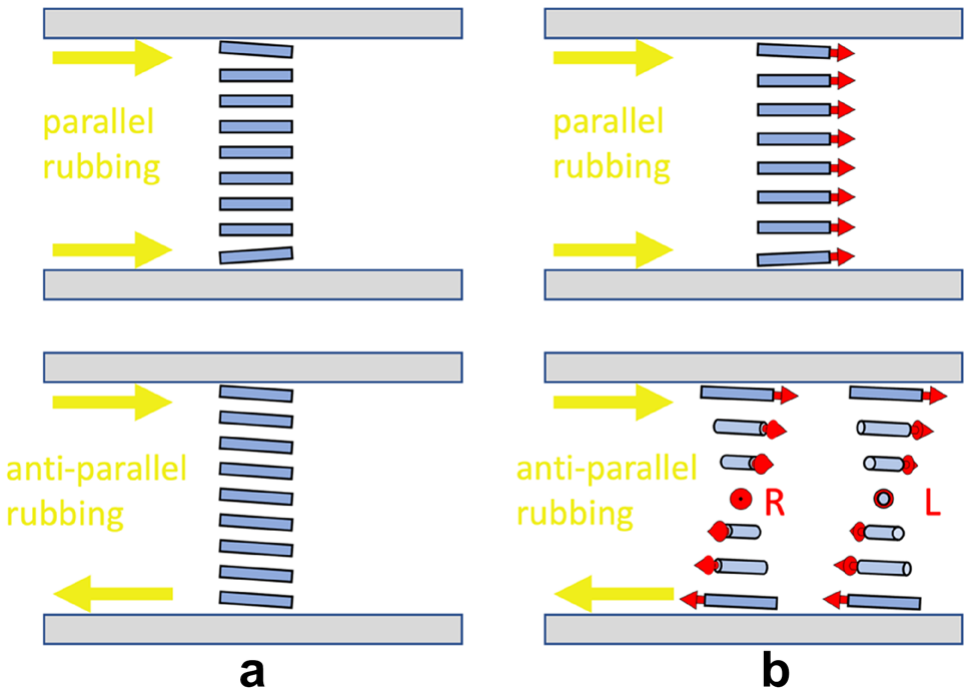
In the N-phase (**a**), parallel and anti-parallel rubbing gives a non-twisted structure. In the N_F_-phase (**b**) the polarization (red) aligns with the rubbing direction. In the case of anti-parallel rubbing the structure has to make a right-handed (R) or left-handed (L) twist of 180° to match the in-plane polar boundary conditions^9,10^.

In a nematic liquid crystal display cell, the quiescent state is governed by the boundary conditions at the two cell substrates. For homogeneous planar anchoring of **n** mechanically rubbed polyimide layers can be used, forcing **n** to align with the rubbing direction at the surfaces. In the conventional N phase, parallel, as well as antiparallel, rubbing at the two surfaces produce a non-twisted director field, Fig. 2a, and if the rubbing directions are not colinear we get a director twist between the plates.

For the polar N_F_ phase the situation is fundamentally different: on a rubbed surface the N_F_ phase aligns with the spontaneous polarization parallel to the rubbing direction^9^. The striking consequence is that when the rubbing directions are anti-parallel, we get a ± 180° director twist between the surfaces, cf. Figure 2b. When the angle between the two rubbing directions is neither 0 nor 180°, the N_F_ phase should adopt the state with the smallest twist matching the rubbing directions. When the two surfaces are rubbed in parallel we get a non-twisted state (just as in the N phase). The possible twisted states and electrooptic effects in the polar nematic phase are further discussed by Caimi et al.^10^ and Sebastián et al. ^11^.

Let us now make a cell where the bottom surface is linearly rubbed, and the top surface is circularly rubbed. Such circularly rubbed cells (CRCs) have been used by Suh et al. to measure the pitch in long pitch cholesteric materials^12^. Linear alignment is obtained by gently rubbing the polyimide coated surface with a velvet cloth in a single direction. Circular alignment can be realized by putting the polyimide-coated substrate on a spinner chuck and gently press the rubbing cloth towards the spinning substrate for a few seconds. Alternatively, as in this study, a small piece of rubbing cloth can be attached to a flat rotating chuck, that is gently pressed towards to a then fixed substrate. As an alternative to mechanical rubbing, photobuffing^13^ could likely be used provided that the pretilt, necessary for the effective in-plane polar surface anchoring, can be secured also in circular alignment.

**Figure 3:**
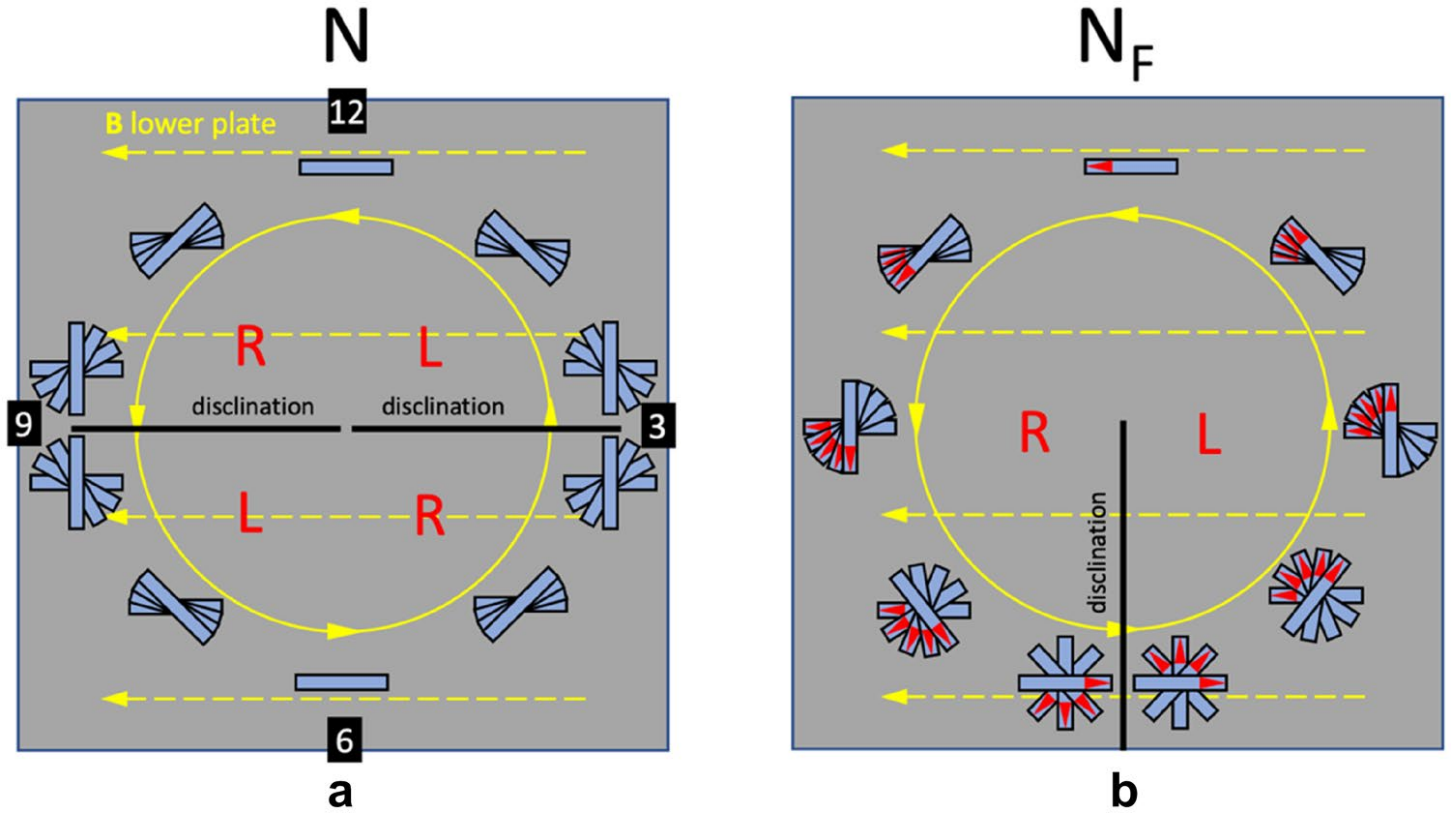
Schematics of circularly rubbed cells filled with (**a**) non polar N, and (**b**) polar N_F_ nematic liquid crystal. The N phase gives a disclination line parallel to the linear rubbing direction **B**, while the N_F_ phase gives a disclination line normal to **B**, starting at the center of circular rubbing. R and L denote right-handed and left-handed twisted regions, respectively.

In a CRC the N phase adopts the configuration illustrated in Fig. 3a^12^. If we use a clockface notation and the bottom substrate is rubbed along the 3–9 direction, we get a non-twisted director field at “12” and “6”. Away from the “12” and “6” positions we get an increasing twist and at 3 and at 9 the twist changes sign, to minimize the twist elastic energy. The discontinuous change from + 90° to − 90° twist occurs across two sharp disclination lines running horizontally, i.e. in the “9”–“3” orientation through the center of circular rubbing.

Let us now consider the case of the N_F_ phase in the same cell, cf. Fig. 3b. The circular rubbing is made counterclockwise, and the rubbing directions are therefore parallel at “12” and antiparallel at “6”. The polar N_F_ phase orients with the polarization along the rubbing directions and to match the boundary conditions, we expect a non-twisted director at “12” where the rubbing directions are in parallel. When going away from “12” towards “6”, clockwise or anticlockwise, there is a twist that increases continuously all the way to the “6” position, where the rubbing directions become perfectly antiparallel. To minimize the elastic energy, the twist discontinuously changes from 180° right-handed to 180° left-handed at “6” across a disclination line, while the structure is continuous at all other positions in the cell. To summarize: in the CRC cell the N phase should make *two disclination lines along the linear rubbing direction*, while the N_F_ should make *one disclination line normal to the linear rubbing direction*, starting from the center of rubbing.

**Figure 4:**
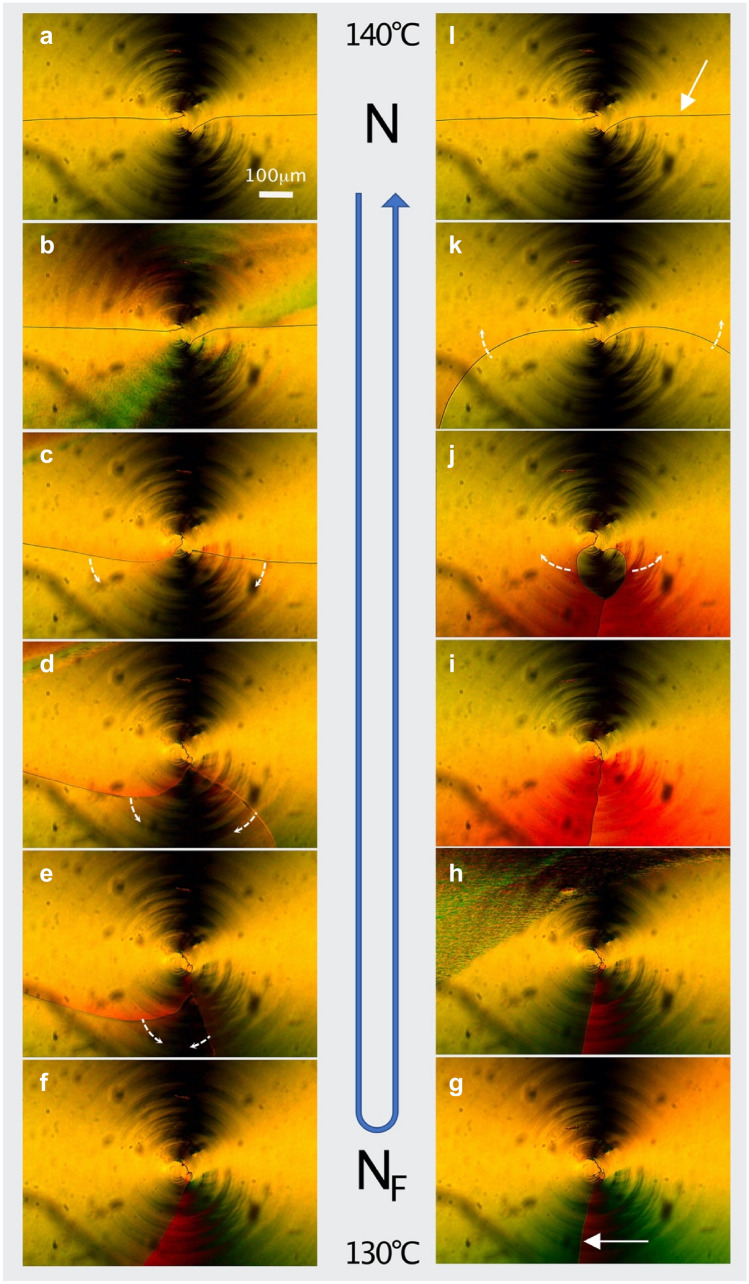
Evolution of the CRC structure of RM734 on cooling from 140 °C to 130 °C (**a**–**f**) and on heating from 130° to 140 °C (**g**-**l**). The disclinations and their direction of motion are marked with solid and dashed arrows, respectively. In **b** and **h**, the regions with irregular texture appear during the phase transition which occurs at approximately the same temperature (at ¨133 °C) on both cooling and heating. The faint circular stripes are due to scratches in the alignment layer, caused by mechanical unevenness during the circular rubbing process. Hence, these stripes visualize the rubbing orientation on the circularly rubbed top plate.

Figure 4 shows polarized optical microscope photographs of a 4 µm thick CRC filled with the archetype N_F_ material RM734 having a phase transition between the ferroelectric N_F_ and the conventional N phase at about 133 °C, and between N and isotropic at 188 °C, respectively. The polyimide aligning layer on both surfaces is PI2610 from DuPont, and the bottom linearly rubbed substrate is the warmer substrate (in direct contact with the hot- plate). The cell was capillary filled with RM734 in the isotropic phase at 190 °C. At 140 °C (**a** and **l**)) we have two horizontal disclination lines emanating from the center of circular rubbing, as expected from the non-polar N phase. At “12” and “6” on the clockface we get extinction of light as the director here is parallel to one of the crossed polarizers. At 130 °C (**f**) the two horizontal disclination lines have been replaced by one disclination line at “6” which proves that the sample is now in the N_F_ phase, cf. Fig. 3. Figure 4a–f shows the evolution of the cell on cooling from 140° to 130 °C at a rate of 3 °C/min. Soon after the phase transition (**b**) the two disclination lines at “9” and at “3” start to move downwards and become one disclination at “6”. On heating from 130 to 140 °C (Fig. 4g–l) the single disclination line at “6”, soon after the transition to the N phase (**h**), splits close to the center of circular rubbing and branches symmetrically into the two horizontal disclinations characteristic of the N phase at the “9” and “3” positions (**l**). The course of events shown in Fig. 4 is perfectly reversible.

The observed defect behavior at the N-N_F_ transition in CRC cells described here can be compared to the behavior of chiral N_F_ materials in so-called Cano-wedge cells ^14,15^. In chiral nematics (N*) the director forms a spontaneous twist with a helix axis normal to the director. In a wedge cell with homogenous planar anchoring, a periodic set of disclination lines appears, normal to the opening direction of the wedge. At each disclination the number of half-pitches increases in steps of one to minimize the elastic energy. In a polar and chiral N_F_* phase where **n** ≠ − **n**, the number of half-pitches instead increases by two in each disclination in such wedge cells^14,15^^.^ Hence the number of disclination lines are then reduced by a factor of 2 at the transition between N* and N_F_*. However, in the Cano-wedge case the material must be *chiral* to show this defect behavior. Furthermore, if the material does not exhibit both the N* phase *and* the N_F_* phase in the sequence, one cannot, merely based on the number of disclination lines in the wedge, draw any conclusion about the polarity of the nematic phase under study. In contrast, the CRC method described here works for both achiral *and* chiral versions of the N_F_ phase, and importantly, regardless of whether the N phase is present or not in the phase sequence. Hence, in liquid crystals with a direct phase transition from isotropic to ferroelectric nematic, see for instance Manabe et al.^16^ and Li et al.^17^, the configuration with one disclination line should still form in CRC cells, revealing the polar order of the N_F_ phase. It is also worth pointing out that the CRC cell method should in principle facilitate pitch measurements of the chiral N_F_* phase, where the pitch in can be calculated from the azimuthal rotation of the single disclination line away from the “6” position, analogous to the pitch measurements of the conventional N* phase in CRC described by Suh et al.^12^.

To conclude, circularly rubbed cells and the position of the disclination line(s) in relation to the linear rubbing direction constitute a simple and yet powerful tool to probe the polar order of nematic liquid crystals, and in particular to unambiguously distinguish the N_F_ phase from the N phase.

The data that supports the findings of this study are available within the article.
